# Functional divergence of LhcSR and PsbS in zeaxanthin‐mediated non‐photochemical quenching

**DOI:** 10.1111/nph.71176

**Published:** 2026-04-11

**Authors:** Claudia Beraldo, Cleo Bagchus, Dana Verhoeven, Alessandra Bellan, Caterina Gerotto, Emilie Wientjes, Herbert van Amerongen, Tomas Morosinotto, Alessandro Alboresi

**Affiliations:** ^1^ Department of Biology University of Padova Via Ugo Bassi 58B Padova 35121 Italy; ^2^ Laboratory of Biophysics Wageningen University 6708 WE Wageningen the Netherlands; ^3^ National Biodiversity Future Center Palermo 90133 Italy

**Keywords:** non‐photochemical quenching, photoprotection, photosynthesis, xanthophyll cycle, zeaxanthin

## Abstract

- To protect photosystem II from excess light, non‐photochemical quenching (NPQ) dissipates excess energy as heat. NPQ relies on trigger proteins, LhcSR in algae and PsbS in vascular plants, and the light‐regulated xanthophyll cycle, which interconverts violaxanthin and zeaxanthin through the opposite activities of violaxanthin de‐epoxidase and zeaxanthin epoxidase (ZEP). Despite extensive research, the molecular mechanisms and the differences in NPQ triggers across lineages remain unclear.
- In this study, we used the moss *Physcomitrium patens*, an evolutionary intermediate possessing both LhcSR and PsbS, to dissect their contributions to zeaxanthin‐mediated quenching by the application of *in vivo* fast spectroscopy analysis.
- In the *zep* knockout (KO) mutant, constitutive zeaxanthin accumulation poises the photosynthetic apparatus in a pre‐quenched state even in the dark, resulting in sustained NPQ upon illumination. Multiple *zep* KO mutants reveal that this constitutive zeaxanthin‐driven quenching is mediated by LhcSR, while PsbS‐dependent quenching is strictly light‐activated.
- Our findings show that the two NPQ triggers, PsbS and LhcSR, have distinct molecular mechanisms suggesting that the evolutionary shift toward PsbS dominance in vascular plants reflects the need for tighter and more energy‐efficient photoprotection.

## Introduction

Photosynthetic organisms rely on sunlight as their primary energy source. Two photosystems (PS) use sunlight to fuel electron transport that produces NADPH and generates a proton motive force across the thylakoid membrane, which drives the synthesis of ATP in the stroma. PSII comprises two functional domains: (1) the light‐harvesting complexes (Lhc), which contain chlorophylls (Chls) and carotenoids that absorb light energy and transfer it to (2) the reaction center, where specialized Chl*a* molecules (P680) drive charge separation. Upon excitation to an excited state (P680*), P680 transfers an electron to a nearby pheophytin molecule, initiating linear electron transport. The oxygen‐evolving complex enables the stepwise extraction of electrons from water, ultimately reducing P680^+^, a highly oxidizing species. This process drives oxygen evolution. Excessive light exposure can lead to the saturation of electron flow, resulting in the formation of reactive oxygen species which can damage PSII leading to photoinhibition and reduced photosynthetic efficiency (Li *et al*., 2009). Photosynthetic organisms have evolved a range of photoprotective strategies that act across different timescales, modulating photosynthetic reactions in response to environmental conditions (Rochaix, 2014). One of them is non‐photochemical quenching (NPQ), which dissipates excess excitation energy as heat through processes occurring in Lhcb and PSII core complexes (Nicol *et al*., 2019). NPQ is activated by acidification of the thylakoid lumen, leading to protonation of specific trigger proteins, LhcSR in green algae and PsbS in vascular plants (Li *et al*., 2002; Peers *et al*., 2009). Another key process is the xanthophyll cycle, which interconverts violaxanthin (Vx) and zeaxanthin (Zx) through the sequential actions of violaxanthin de‐epoxidase (VDE) and zeaxanthin epoxidase (ZEP) (Arnoux *et al*., 2009; Goss & Jakob, 2010; Jahns & Holzwarth, 2012). Under excess light, lumen acidification activates VDE, converting Vx into Zx and promoting thermal energy dissipation. NPQ can be divided into several components that differ in activation kinetics and regulation, including energy‐dependent quenching (qE) (Pascal *et al*., 2005), a slowly reversible type of NPQ taking place in the antenna of PSII (qH) (Malnoë *et al*., 2018), the zeaxanthin‐dependent quenching (qZ) (Nilkens *et al*., 2010) and the photo‐inhibitory quenching (qI) (Walters & Horton, 1993; Malnoë, 2018). qE is dependent on (1) thylakoid lumen acidification, (2) protonation‐induced conformational changes in LhcSR and PsbS (Li *et al*., 2002; Peers *et al*., 2009) (3) Vx‐to‐Zx conversion (Niyogi *et al*., 1998). While qZ induction is pH dependent, qZ persists longer than qE and its relaxation is largely independent of pH. When plants return to limiting light conditions, qZ relaxes over a period of 10–60 min, in parallel with the reconversion of Zx to Vx catalyzed by ZEP (Kress & Jahns, 2017). This persistence allows plants to respond more rapidly to subsequent episodes of excess light (Kromdijk *et al*., 2016; De Souza *et al*., 2022). In addition, Zx can directly quench singlet oxygen, mitigating oxidative stress (Havaux *et al*., 2004). The qZ component has been proposed to arise from the binding of Zx to trimeric LHCII (trimeric Lhcb1/2/3) (Johnson *et al*., 2009; Xu *et al*., 2015) and monomeric Lhcb antenna proteins, such as Lhcb5 (CP26) (Dall'Osto *et al*., 2005), Lhcb4 (CP29) and Lhcb6 (CP24) (Johnson *et al*., 2009) in *Arabidopsis thaliana* (Sylak‐Glassman *et al*., 2014; Son *et al*., 2020). Finally, qI refers to the photoinhibition‐related quenching; it occurs within the PSII core and involves processes that make it the slowest component of NPQ.

Although the primary components involved in NPQ activation (i.e. LhcSR, PsbS, and Zx) have been identified, their precise molecular interactions and individual contributions to energy dissipation remain only partially understood. In addition, the existence of two distinct protein triggers across the green lineage suggests an evolutionary divergence in NPQ regulation. LhcSR, which binds pigments including Zx, has been shown *in vitro* to mediate quenching regulated by pH and by Zx, as demonstrated using isolated LhcSR3 from *Chlamydomonas reinhardtii* (Bonente *et al*., 2011; Liguori *et al*., 2013; Troiano *et al*., 2021) and LhcSR1 from *Physcomitrium patens* (Kondo *et al*., 2017; Pinnola *et al*., 2017). By contrast, PsbS does not bind pigments but enhances the quenching capacity by sensing low lumenal pH and potentially inducing conformational changes in nearby Lhcs (Niyogi & Truong, 2013; Fan *et al*., 2015; Son *et al*., 2020). Experiments with reconstituted PsbS, LHCII, and Zx in proteoliposomes showed that while both PsbS and Zx promote LHCII aggregation, neither alone was sufficient on its own to induce effective quenching (Wilk *et al*., 2013).

In this study, we used the moss *Physcomitrium patens* to investigate the molecular mechanisms underlying Zx‐induced quenching. This system offers a unique opportunity to study simultaneously, for the first time *in vivo*, the constitutive interactions between Zx, LhcSR, and PsbS, as *P. patens* possesses both NPQ triggers as well as an active xanthophyll cycle (Alboresi *et al*., 2010; Pinnola *et al*., 2013; Beraldo *et al*., 2023). In particular, the genome of *Physcomitrium patens* carries two copies of *LhcSR* and a single copy of *PsbS* genes (Alboresi *et al*., 2008). The LhcSR1 isoform is more abundantly expressed than LhcSR2 and provides the major contribution to NPQ (Alboresi *et al*., 2010; Gerotto *et al*., 2011). Moreover, unlike *C. reinhardtii*, where LhcSR accumulation requires exposure to excess light in minimal medium (Bonente *et al*., 2011), *P. patens* LhcSR proteins are already present under control light conditions (Alboresi *et al*., 2010). Furthermore, this moss possesses multiple copies of major LHCII components shared with both green algae and seed plants, as well as minor antenna complexes Lhcb4, Lhcb5, and Lhcb6 (Alboresi *et al*., 2008). We took advantage of the possibility to generate multiple knockout (KO) mutants, and the results showed that *zep* KO plants, which completely lack the ZEP enzyme and accumulate Zx even under low‐light conditions, exhibit a constitutive quenching. The generation of *zep lhcsr* KO and *zep psbs* KO mutants revealed that the quenching conformation in the dark depends on LhcSR and not on PsbS, which dissipate excess energy only when light is present. Overall, these results offer new insights into the different molecular mechanisms of LhcSR and PsbS in modulating NPQ and suggest that PsbS does not provide a stronger quenching ability but rather offers a more tightly regulated mechanism for energy dissipation.

## Materials and Methods

### Plant materials and growth conditions

Protonemal tissue of *Physcomitrium patens*, Gransden WT strain, *zep* KO (Takezawa *et al*., 2015), *psbs* KO (Alboresi *et al*., 2010), *lhcsr* KO (Alboresi *et al*., 2010), *psbs lhcsr* KO (Alboresi *et al*., 2010), *zep psbs* KO, *zep lhcsr* KO and *zep psbs lhcsr* KO lines were grown under controlled conditions: 22°C, 16 h : 8 h, light : dark. For physiological characterization, plants were grown for 10–11 d at 50 μmol photons m^−2^ s^−1^ on minimum medium (PPNO_3_) (Ashton *et al*., 1979), while plant propagation for maintenance was performed on rich medium (PPNH_4_) (Ashton *et al*., 1979).

### Chlorophyll and carotenoid analysis

Small pieces of 2–5 mm in diameter of protonema tissue were frozen in liquid nitrogen and ground using pestles for 1.5‐ml microcentrifuge tubes. Pigments were extracted in 0.5 ml of 85% acetone and analyzed by high‐performance liquid chromatography (HPLC) after two steps of centrifugation at maximum speed for 15 min at 4°C (Gilmore & Yamamoto, 1991).

### *In vivo* Chl fluorescence measurements

NPQ phenotype was assessed by *in vivo* Chl fluorescence signal monitored at room temperature with a Dual PAM‐100 fluorometer (Walz) in protonemal tissues grown for 10 d in PPNO_3_ medium. Before measurements, plants were dark‐adapted for 40 min. For induction/recovery kinetics, actinic light 850 μmol m^−2^ s^−1^ photons was used (saturating actinic light). PSII parameters were calculated as follows: *F*_v_/*F*_m_ as (*F*_m_ – *F*_0_)/*F*_m_, NPQ as (*F*_m_ − *F*_m_′)/*F*_m_′. Fluorescence traces in Fig. 2 (see later) were normalized to *F*_m_.

### Moss transformation and mutant selection

*zep* KO line 2 (Takezawa *et al*., 2015) was used as genetic background to obtain *zep psbs* KO, *zep lchsr1* KO lines. *LhcSR1* (locus XM_001776900) and *PsbS* (locus XM_001778511) coding sequences were knocked out by homologous recombination‐mediated gene targeting as previously described (Alboresi *et al*., 2010). The disruption of *PsbS* and *LhcSR1* gene was verified by RT‐PCR (Supporting Information Figs S2, S3, see later) and immunoblot analysis confirmed the absence of protein accumulation in two independent *zep psbs* KO and *zep lhcsr* 1 KO lines that were retained for subsequent analysis (Figs 4a, S3, see later). We next applied Crispr Cas9 technology (Guyon‐Debast *et al*., 2021) to knockout *LhcSR2* (locus XM_001768019) *in zep lhcsr1* KO and *zep psbs lhcsr* 1 KO (Figs S4, S5, see later). Two single guide RNAs (sgRNAs) were designed to target the two exons of *lhcsr2* in conjunction with Cas9 (Fig. S4; Table S1). After transient selection on antibiotic‐containing medium and subsequent regeneration, the transfected plants were genotyped by PCR (Figs S4, S5, see later). In *zep psbs* KO background the *LhcSR2* gene in a subset of the regenerated plants (*c*. 80% of the total) displayed a WT‐like size, while another subset (*c*. 20% of the total) exhibited large deletions. All PCR products were sequenced for confirmation, and lines #1 and #2 were selected for further analysis. Specifically, line #1 exhibited a 4 bp deletion at the beginning of the second exon, while line #2 showed a 559 bp deletion encompassing the first exon and the first intron (Fig. S4, see later). RT‐PCR revealed truncated transcript in line #2, while the absence of transcript in line #1 and immunoblot analysis revealed lack of LhcSR2 accumulation in both selected lines (Fig. S4, see later, Fig. 4b). Starting from the *zep psbs lhcsr 1* KO background, Crispr Cas9 technology allowed the isolation of two independent zep *psbs lhcsr 1 lhcsr 2* KO lines, hereafter referred to as zep *psbs lhcsr* KO for simplicity. Line #1 exhibited a 559 bp deletion in the first exon and in the first intron, while line #2 accumulated several missense mutations within the first 560 bp of the *LhcSR2* gene (Fig. S5, see later). RT‐PCR and immunoblot analysis confirmed the lack of *LhcSR2* transcript and LhcSR2 protein (Figs S5, 4c see later). All primers employed in this work are listed in Table S2.

### Immunoblot analysis

Total protein extracts from *P. patens* were prepared by grinding tissues in sample buffer before SDS‐PAGE. For immunoblot analysis, proteins were transferred onto nitrocellulose membranes and detected using specific homemade polyclonal antibodies. Primary antibodies against *P. patens* PsbS (α‐PsbS), LhcSR (α‐LhcSR), and D2 (α‐D2) were used at dilutions of 1 : 1000, 1 : 40 000, and 1 : 1000, respectively. For D2, a mixture of barley, spinach, and Synechocystis D2 proteins was used as antigen (Barbato *et al*., 1992). Polyclonal antibodies were raised in rabbits against full‐length proteins expressed in *Escherichia coli*. Signals were detected using an anti‐rabbit secondary antibody conjugated to alkaline phosphatase (Cat. #31340; 1 : 10 000; Merck, Darmstad, Germany), and colour development was performed with NBT/BCIP substrates according to standard procedures.

### Streak camera measurements

Chl quenching in the *zep* KO mutants of *P. patens* was measured with a streak‐camera setup (Van Stokkum *et al*., 2008). With the current system, picosecond fluorescence results were obtained, as described in previous studies (Farooq *et al*., 2018; Bos *et al*., 2019). The system consists of a pulsed laser source (Rock white light laser; Leukos, Limoges, France) and a streak‐camera detecting system (C10910; Hamamatsu, Hamamatsu City, Japan). Measurements were performed on *P. patens* at room temperature. Petri dishes with protonema (10–15 d old) were placed in a circular cuvette. This circular cuvette was rotated at 3 rpm and moved horizontally at 1 rpm to prevent long laser exposure, which can induce photodamage and singlet‐triplet annihilation (Chukhutsina *et al*., 2019).

Chls and carotenoids in the moss were excited by focusing the pulsed laser (488 ± 10 nm and 38 MHz) on the protonema inside the rotating cell, below the rotational axis. Chl fluorescence emission was collected above 640 nm (to remove scattered excitation light and actinic light) by a spectrograph with a grating of 150 g mm^−1^ (Shamrock, Andor, Belfast, UK) to generate a spectrum for the streak camera. The time window to collect fluorescence was *c*. 3 ns. Measurements were collected with an integration time of 1 s over at least 450 frames. To close the PSII reaction centres, mosses were incubated with a DCMU buffer (30 μM DCMU, 150 mM sorbitol and 10 mM Hepes) for at least 45 min in the dark.

### Data analysis streak camera measurements

The resulting spectra were corrected for the background and the wavelength‐dependent sensitivity of the detector. These corrected data were analyzed with the R‐package timp based software Glotaran (Snellenburg *et al*., 2012). The analysis results in decay‐associated spectra (DAS), showing an emission spectrum for every fluorescence lifetime component in the streak image. The DASs are normalized to the same total area under the curves.

### Determination of corrector factors for the zep KO mutants

As suggested by the decrease in PSII fluorescence lifetime in the dark when all RCs are closed, the *zep* KO mutants exhibit dark‐adapted quenching, which reduces the apparent NPQ and *F*_v_/*F*_m_ values measured by PAM fluorometry. Without dark‐adapted quenching, the *zep* KO mutants are assumed to reach WT *F*_m_. A correction factor, based on the non‐quenched WT *F*_m_, can thus adjust for the lowered *F*_m_ caused by this dark quenching in the mutants:

$$F_{m}^{\mathrm{No}\;\mathrm{NPQ}}=F_{m}^{\text{Observed}}\left[\frac{\tau_{F_{m}}^{\mathrm{No}\;\mathrm{NPQ}}}{\tau_{F_{m}}^{\text{Observed}}}\right]$$

where $F_{m}^{\mathrm{No}\;\mathrm{NPQ}}$ is the estimated value of the dark‐adapted fluorescence level with fully closed PSII RCs and no NPQ, $F_{m}^{\text{Observed}}$ is the measured value of the dark‐adapted fluorescence level with fully closed PSII RCs with long‐term quenching (as in the *zep* KO mutants), $\tau_{F_{m}}^{\mathrm{No}\;\mathrm{NPQ}}$ is the theoretical value of the average dark‐adapted fluorescence lifetime with fully closed PSII RCs and no NPQ (assumed to be the same as in WT plants) and $\tau_{F_{m}}^{\text{Observed}}$ is the measured value of the average dark‐adapted fluorescence lifetime with fully closed PSII RCs with long‐term quenching (as measured in the *zep* KO plants) (Ramakers *et al*., 2025).

To determine the average fluorescence lifetimes necessary for the determination of the correction factor, the data from the streak‐camera measurements on *P. patens* WT and *zep* KO mutants were used. The DUAL‐PAM collects fluorescence above 700 nm due to the presence of an RG‐9 filter. The average fluorescence lifetime measured with the streak camera setup was therefore determined above 700 nm. The contribution of both the PSI and the PSII DAS were determined above this 700 nm cutoff and multiplied by their respective fluorescence lifetime to determine the average fluorescence lifetime required for the correction factor. The resulting correction factor for the *zep* KO mutant was 1.49. This correction factor was used to calculate corrected NPQ as [(*F*_m_ − *F*_m_′)/*F*_m_′] × 1.49 in Fig. S1.

### Statistical analysis

Descriptive statistics and inferential statistics were performed using OriginPro9.1 software©. Differences between genotypes were statistically tested by One‐Way ANOVA. Tukey's Honestly Significant Difference (HSD) *post hoc* test was used to evaluate pairwise differences between genotypes.

## Results

### Zeaxanthin epoxidase enzyme is crucial for xanthophyll biosynthesis

**Fig. 1:**
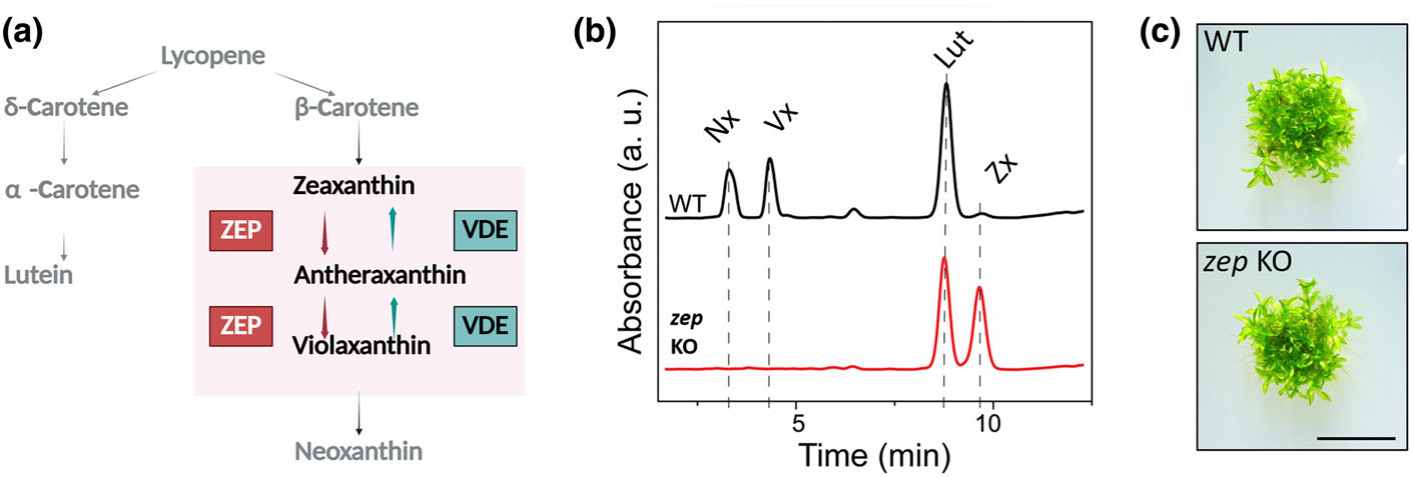
Carotenoid biosynthesis and pigment composition in *Physcomitrium patens* WT and *zep* KO plants. (a) The carotenoid biosynthesis pathway of *P. patens* is depicted with a focus on the photoprotective xanthophyll cycle (pink background). Under low light condition, zeaxanthin is sequentially converted into antheraxanthin and violaxanthin by the enzyme zeaxanthin de‐epoxidase (ZEP). Conversely, under excess light conditions, violaxanthin de‐epoxidase (VDE) reverses this process, catalyzing the conversion of violaxanthin into antheraxanthin and subsequently to zeaxanthin. (b) High‐performance liquid chromatograms of photosynthetic pigments were obtained from protonema of 11‐d old *P. patens* plants. The chromatograms compare WT (upper black line) and *zep* KO (lower red line) samples. The absorbance at 440 nm was monitored over retention time, with labeled peaks corresponding to specific carotenoids: Nx, neoxanthin; Vx, violaxanthin; Lut, lutein; Zx, zeaxanthin. (c) 28 d old WT and *zep* KO plants growth phenotype. Plants were grown under 50 μmol photons m^−2^ s^−1^. Bar is valid for both WT and *zep* KO, 5 mm.

The ZEP enzyme plays a critical role in the carotenoid biosynthesis pathway by catalyzing the conversion of Zx into antheraxanthin and subsequently into Vx (Fig. 1a). To investigate its function in *P. patens*, we used *zep* KO mutant lines. HPLC analysis of pigment profiles revealed that the protonema of *zep* KO mutant accumulated high levels of Zx but lacked Vx and neoxanthin (Fig. 1b), which is produced from Vx. Notably, Zx levels in the *zep* KO exceeded the total xanthophyll pool (Vx + Zx) found in WT plants. Despite these significant alterations in xanthophyll composition, there were no substantial differences between *zep* KO and WT plants in terms of Chl *a*/*b* ratio or total Chl/Car content suggesting a similar composition of photosynthetic apparatus (Table 1). Furthermore, *zep* KO plants exhibited no observable growth defects relative to WT after 28 d of growth under control light conditions (Fig. 1c).

**Table 1:** Changes in Chl nd carotenoid content in protonema cells.

	WT	*zep* KO
Neoxanthin	5.66 ± 0.22	nd
Violaxanthin	5.61 ± 0.73	nd
Lutein	21.17 ± 0.58	18.11 ± 0.07
Antheraxanthin	nd	nd
Zeaxanthin	nd	11.87 ± 0.66
β‐carotene	7.51 ± 0.20	7.56 ± 0.88
Vx + Zx	5.61 ± 0.73	11.87 ± 0.66
Car/*Chl*	0.40 ± 0.01	0.38 ± 0.01
*Chl a*/*b*	2.28 ± 0.03	2.17 ± 0.05

*Physcomitrium patens* plants were dark‐adapted for 45 min directly on minimal agar medium and then used for pigment extraction. Acetone extracts from each sample were analyzed by HPLC to separate and quantify individual pigments. The content of each carotenoid species was expressed as moles per 100 moles of total Chl (*Chl a* + *b*). The xanthophyll cycle pool was calculated as the sum of violaxanthin, antheraxanthin, and zeaxanthin. The *Chl a*/*b* and carotenoid/Chl ratios were estimated by deconvolution of the absorption spectra of the acetone extracts (Chazaux *et al*., 2022). Data are expressed as means ± SD, *n* = 3. nd, not detected. Plants were grown under 50 μmol photons m^−2^ s^−1^.

### Constitutive zeaxanthin accumulation induces quenching in the dark

**Fig. 2:**
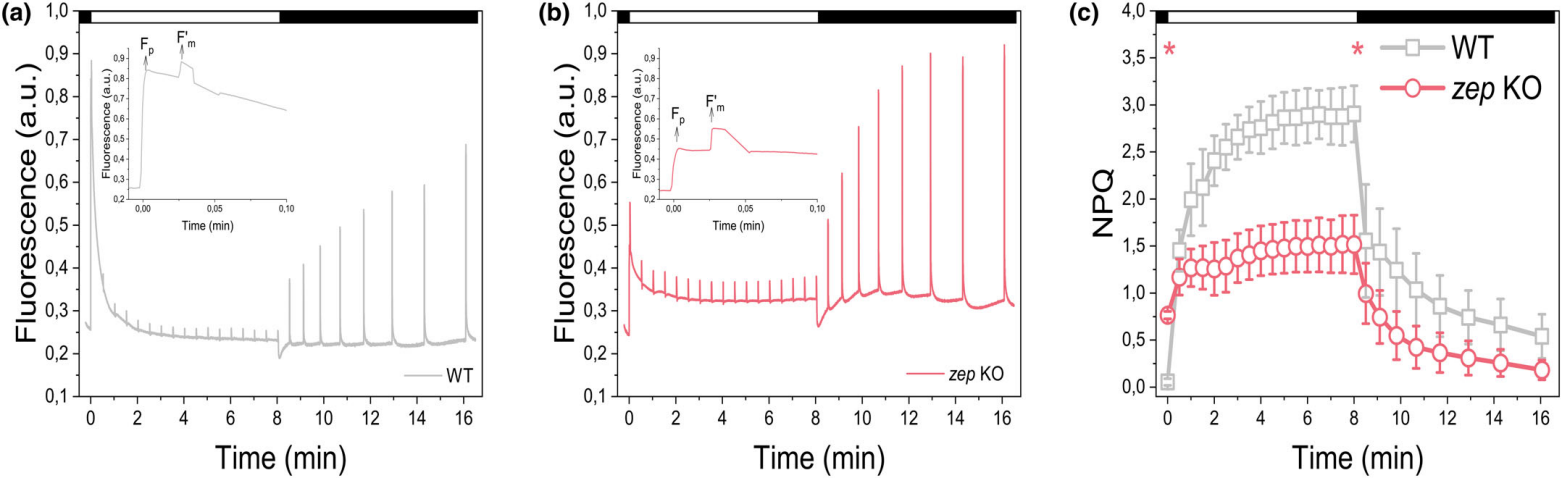
*Physcomitrium patens zep* KO quenching properties. (a, b) Representative Chl*a* fluorescence traces of WT (a) and *zep* KO (b) lines normalized to *F*_m_. Arrows indicate *F*_p_ point, corresponding to fluorescence level at the onset of actinic light exposure. (c) NPQ kinetics of WT (black squares) and *zep* KO (red circles) plants. Exposure to actinic irradiance is indicated at the top of each panel. Actinic light intensity was 850 μmol of photons m^−2^ s^−1^. The first NPQ data point was obtained from a saturating flash delivered 0.1 s after actinic light onset. Data represent mean ± SD from more than four independent biological replicates (*n* > 4). Asterisks indicate significant differences between *zep* KO and WT (one‐way ANOVA, *P* < 0.01). Statistical analysis was performed at the 1‐s and 8‐min timepoints.

We next evaluated the effects of constitutive Zx accumulation on the photosynthetic performance of *P. patens* by measuring Chl*a* fluorescence using PAM fluorometry to assess PSII functionality and NPQ capacity. The *zep* KO mutant exhibited a significantly reduced *F*_v_/*F*_m_ (0.69 ± 0.03), a proxy for maximal quantum efficiency of PSII photochemistry, compared to WT (0.77 ± 0.02). Upon the onset of actinic light, *zep* KO mutants exhibited a pronounced and rapid quenching of Chl*a* fluorescence, clearly exceeding the quenching observed in WT plants (Fig. 2a). In WT, the initial fluorescence level following actinic light exposure (*F*_p_) (Baker, 2008) exceeded 90% of the maximum fluorescence (*F*_m_), whereas in *zep* KO lines, *F*_p_ was only *c*. 55% of *F*_m_ (Fig. 2a). Also, the first measurement of *F*_m_′ showed a strong reduction in zep KO as compared to WT, suggesting the actinic light immediately induced a strong quenching. This phenomenon may also explain the difference in *F*_v_/*F*_m_ between dark‐adapted WT and *zep* KO plants, as 1 − *F*_v_/*F*_m_ reflects the constitutive non‐photochemical quenching of excitation energy in dark‐adapted PSII (Lazár, 2015).

However, although *zep* KO plants exhibited a faster NPQ induction, the overall amplitude remained lower than in WT throughout the actinic light treatment (Fig. 2b).

**Fig. 3:**
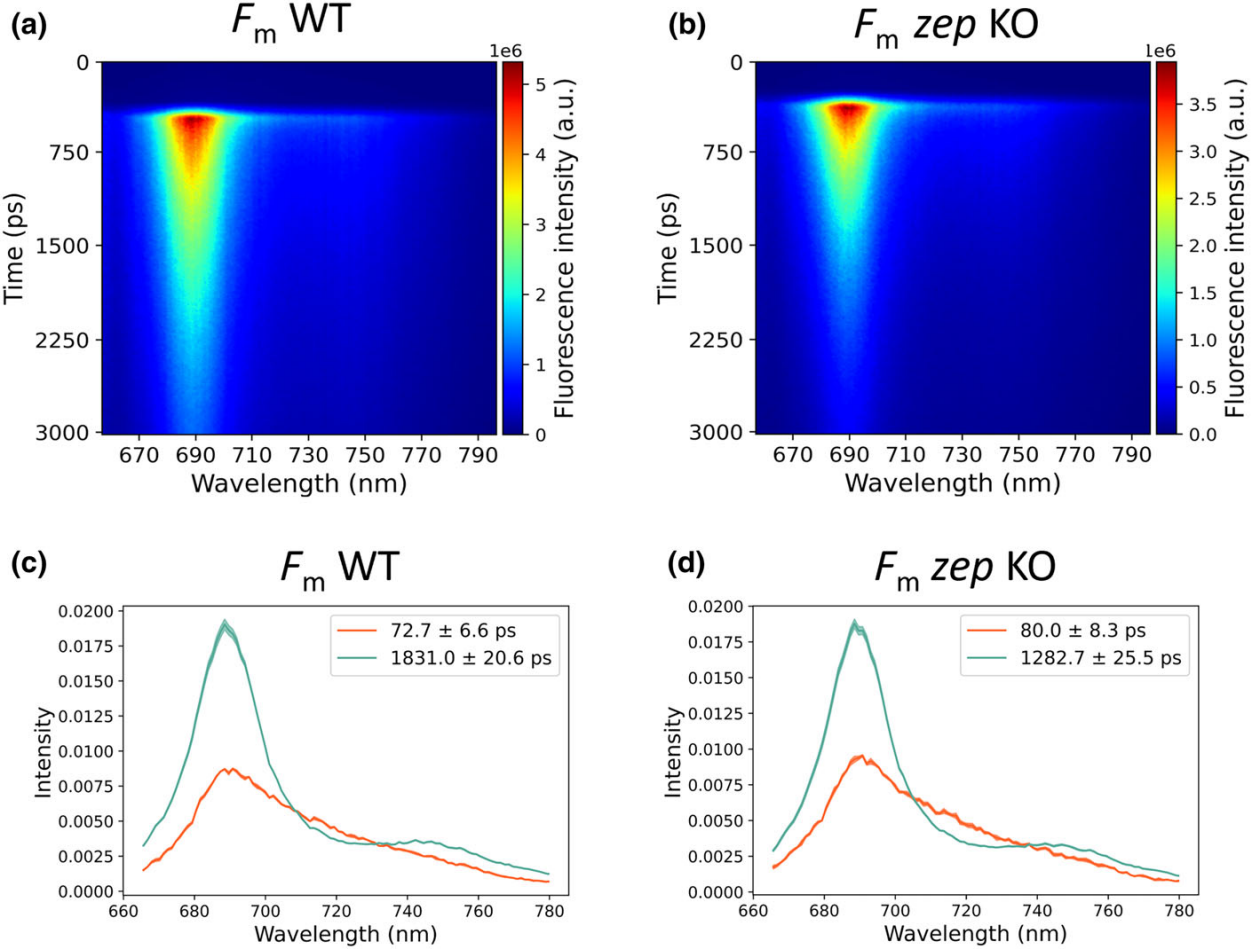
*Physcomitrium patens zep* KO dark‐adapted quenching. (a, b) Representative streak camera images of time‐resolved fluorescence analysis of WT and *zep* KO lines under dark conditions (*F*_m_). (c, d) Two decay‐associated spectra of WT and *zep KO* mutant. Orange is attributed to PSI and green is attributed to PSII. Shading indicates the SD, *n* = 3. Lifetimes ±SD are indicated in the insets (*n* = 3).

Plants were investigated in dark‐adapted state using time‐resolved fluorescence analysis using a streak camera system. Fluorescence decay curves were separated based on the wavelength, resulting in two‐dimensional images with time on the y‐axis (0–3 ns), wavelength on the x‐axis (665–780 nm) and fluorescence intensity as a false color map (Fig. 3). In dark‐adapted plants, treated with DCMU to close the PSII reaction centers, the fluorescence decay was much faster in the *zep* KO compared to the WT (Fig. 3a,b). A global analysis approach was employed to quantify the fluorescence kinetics, generating DAS (Fig. 3c,d). The spectra can be accurately described with two fluorescence lifetime components. Based on their spectra, the longest lifetime (*c*. 1.8 ns in WT) is ascribed to PSII, while the shortest lifetime (*c*. 80 ps) is ascribed to PSI (Wientjes *et al*., 2011). In *zep* KO the same two components were identifiable with spectra similar to WT. The PSII lifetime was significantly shorter in the *zep* KO (*c*. 1.3 ns) than in the WT, while the PSI lifetime did not change. Notably, the PSI emission spectrum of *P. patens* differs from *A. thaliana*, because its PSI antenna system does not contain an antenna subunit equivalent to Lhca4. Consequently, PSI from *P. patens* lacks the strongly red‐shifted emission characteristic of vascular plant PSI (Croce *et al*., 2000; Wientjes *et al*., 2011; Gorski *et al*., 2022; Liu *et al*., 2025).

These results demonstrate that in the dark‐adapted state of *zep* KO plants, PSII is constitutively quenched even in the absence of actinic light and of the subsequent decrease in lumenal pH. This constitutive quenching resulted in a reduction of maximal fluorescence, which explains the lower *F*_v_/*F*_m_ ratio in the *zep* KO compared to the WT. To account for the presence of quenching in dark‐adapted *zep* KO, as reflected by the decreased PSII fluorescence lifetime in the dark, we recalculated NPQ using a correction factor based on the non‐quenched WT *F*_m_ (see the Materials and Methods section for details and Fig. S1). This correction reduced the gap in steady‐state NPQ values (after 6 min) between WT and the *zep* KO, suggesting most of the reduction in light‐induced quenching is due to the dark‐adapted quenching present in the *zep* KO (Fig. S1).

### Zx‐dependent quenching is primary mediated by LhcSR

**Fig. 4:**
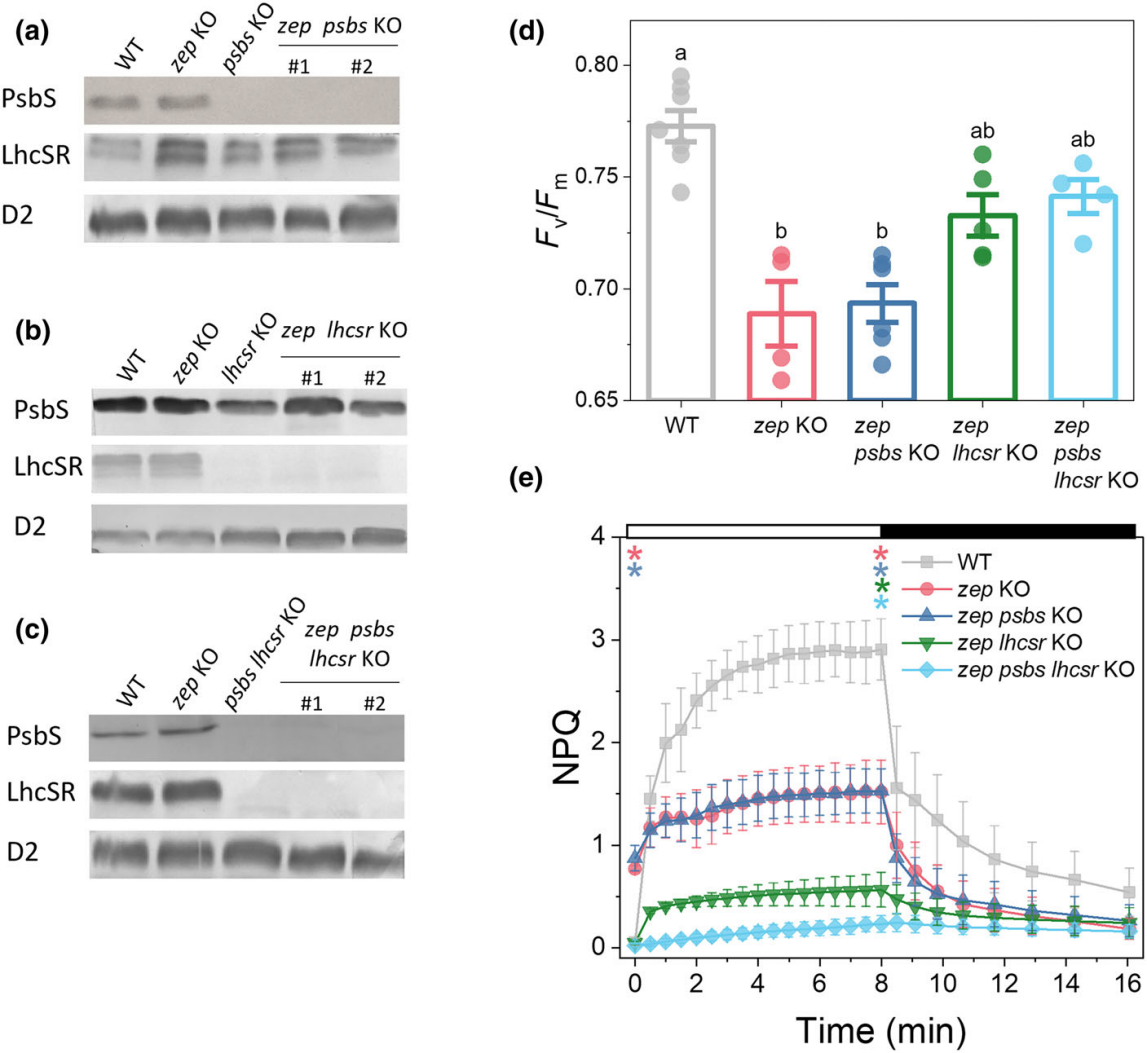
Isolation and characterization of *Physcomitrium patens* *zep psbs*, *zep lhcsr* and *zep psbs lhcsr* KO mutant lines. (a–c) Immunoblot analysis of PsbS, LhcSR, and D2 proteins in WT, *zep* KO, *zep psbs* KO, *zep lhcsr* KO, and *zep psbs lhcsr* KO mutants. (d) *F*_v_/*F*_m_ of *zep* mutant lines. Values marked with different letters are significantly different (one‐way ANOVA followed by Tukey's Honestly Significant Difference (HSD) *post hoc* test, *P* < 0.01). (e) NPQ of *zep* mutant lines. A 10 d old protonema was treated with 850 μmol of photons m^−2^ s^−1^ of actinic light followed by 8 min of dark. WT (black), *zep* KO (red) *zep psbs* KO (blue), *zep lhcsr* KO (green), and *zep psbs lhcsr* KO (light blue). Data represent mean ± SD. Asterisks indicate significant differences between WT and *zep* or between WT and *zep trigger* KO (*psbs*, *lhcsr*, or *psbs lhcsr*) (one‐way ANOVA followed by Tukey's HSD *post hoc* test, *P* < 0.05). Timepoint 1 s and 8 min were considered for statistical analysis. See Supporting Information Table S4 for detailed statistical results.

*Physcomitrium patens* NPQ has the unique characteristic of being induced by two distinct classes of Lhc‐like proteins, namely PsbS and LhcSR (Alboresi *et al*., 2010). To investigate how these two triggers regulate NPQ in the presence of constitutive zeaxanthin accumulation, we knocked out PsbS and/or LhcSR in the *zep KO* background. Since the *P. patens* genome contains two LhcSR genes, to obtain a complete *lhcsr* knockout in the *zep* background, we used a two‐step approach: first isolating *zep lhcsr1*, followed by the *zep lhcsr1 lhcsr2* double mutant, hereafter referred to as *zep lhcsr KO* (see the Materials and Methods section and Figs S2–S5 for details). We generated at least two independent lines for each genotype: *zep psbs KO*, *zep lhcsr KO*, and *zep psbs lhcsr KO*. Knockout lines were validated by the absence of transcripts (RT‐PCR, Figs S2–S5) and accumulated protein via immunoblot analysis (Fig. 4a–c). Additionally, immunoblot analysis revealed that no difference was observed in the accumulation of D2, chosen as representative PSII subunit, between WT, *zep* KO and *zep* KO crossed with different trigger mutations (i.e. *psbs* and *lhcsr* KO). WT and *zep* KO exhibited similar PsbS and LhcSR content. Similarly, LhcSR levels were comparable between *zep* KO and *zep psbs* KO (Fig. 4a), and PsbS levels were unchanged between *zep* KO and *zep lhcsr* KO (Fig. 4b).

The *zep psbs KO* plants exhibited lower *F*_v_/*F*_m_ (0.69 ± 0.02) compared to WT, with values similar to the *zep KO*. By contrast, *F*_v_/*F*_m_ was slightly higher in *zep lhcsr* KO (0.73 ± 0.02) and *zep psbs lhcsr* KO (0.74 ± 0.02) than in *zep* KO, though none of the *zep* mutant lines reached WT levels (Fig. 4d). As concluded for *zep* KO, this decrease in *F*_v_/*F*_m_ is likely attributable to constitutive Zx accumulation, rather than the absence of PsbS, LhcSR, or both (Table S3), as the *psbs* KO (0.78 ± 0.03), *lhcsr* KO (0.77 ± 0.02), and *psbs lhcsr* KO (0.78 ± 0.02) lines, lacking these proteins but not ZEP, maintained *F*_v_/*F*_m_ values closer to WT.

The *zep psbs* KO plants exhibited elevated NPQ compared to WT at the onset of actinic light but afterwards they showed no significant difference with *zep* KO and WT plants (Figs 4e, S6). Conversely, the *zep lhcsr KO* line displayed reduced NPQ at the onset of actinic light exposure compared to *zep KO*, close to WT level (Fig. 4e). At steady‐state, *zep lhcsr KO* plants exhibited lower NPQ than *zep* KO (Fig. 4e), close to the levels of *lhcsr* KO plants (Fig. S6). These results indicate that LhcSR proteins are the primary mediators of Zx‐driven quenching in *P. patens*. Finally, *zep psbs lhcsr* quadruple mutants showed no residual qE capacity (Figs 4e, S6), confirming that both Zx and LhcSR are required for the full expression of energy‐dependent quenching in the absence of PsbS.

**Fig. 5:**
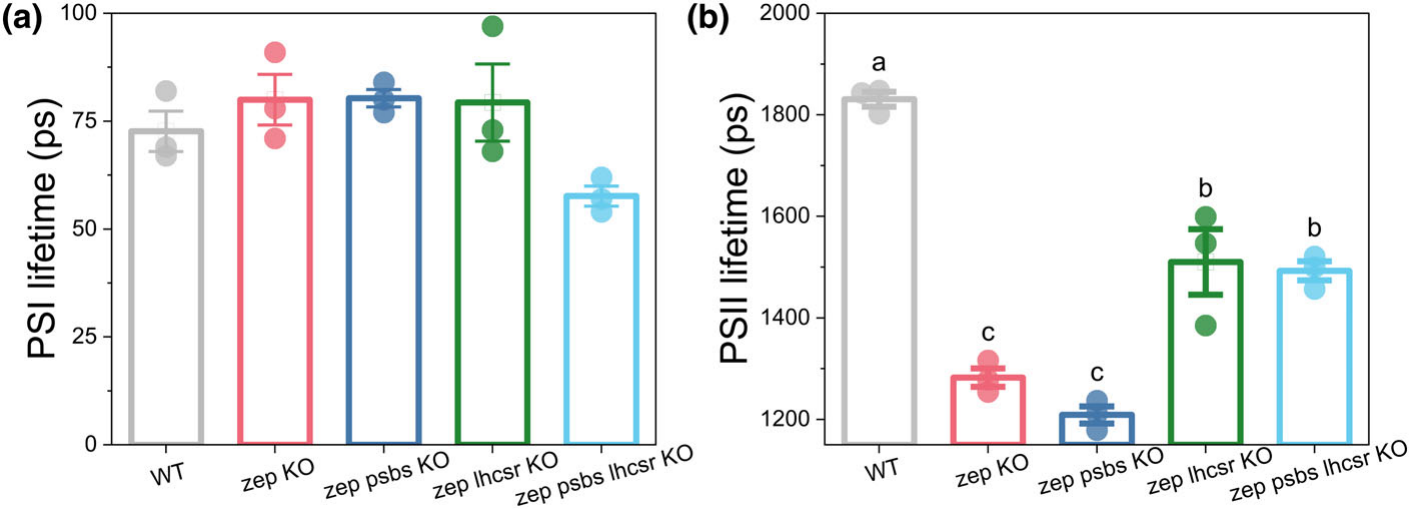
PSI (a) and PSII (b) lifetimes of multiple *Physcomitrium patens* *zep* mutants under dark conditions. *zep* KO (red) *zep psbs* KO (blue), *zep lhcsr* KO (green), and *zep psbs lhcsr* KO (light blue). Data represent mean ± SE (*n* = 3). Individual measurement points are shown as dots. (a) One‐way ANOVA (*P* < 0.01) indicated no significant difference between groups, (b) values marked with different letters are significantly different (one‐way ANOVA followed by Tukey's Honestly Significant Difference (HSD) *post hoc* test, *P* < 0.01).

Due to the different behaviors observed during dark‐to‐light transitions among the *zep* single and *zep* mutants crossed for trigger mutations, we extended our analysis of Chl fluorescence lifetimes to include all combinations of *zep* KO lines under dark‐adapted conditions, as previously done for single *zep* KO line (Figs 5, S5). While PSI‐associated fluorescence lifetimes remained consistent across all genotypes (Figs 5a, S7), significant differences were detected in PSII fluorescence lifetimes (Figs 5b, S7). This analysis revealed three distinct groups: (1) WT plants exhibited the longest PSII fluorescence lifetimes, consistent with an unquenched state, (2) *zep KO* and *zep psbs KO* mutants showed the shortest lifetimes, indicative of a quenched state, (3) *zep lhcsr KO* and *zep psbs lhcsr KO* mutants displayed intermediate lifetimes, statistically indistinguishable from one another, and falling between the WT and *zep* KO groups. These findings agree with the *F*_v_/*F*_m_ measurements (Fig. 4) and suggest that the enhanced quenching observed in the *zep* KO background is driven by the synergistic action of zeaxanthin Zx and LhcSR. By contrast, PsbS does not contribute to the dark‐quenched state.

## Discussion

Over the past few decades, key components of NPQ have been identified by genetic analysis and mutants isolation (Li *et al*., 2009; Niyogi & Truong, 2013), yet a comprehensive understanding of the underlying mechanisms and the interplay between involved proteins remains incomplete. These data also showed a diversity of NPQ molecular mechanisms that show significant differences across species, starting from the different molecular triggers that are LhcSR/LhcX in most eukaryotic algae, while plants rely on PsbS (Niyogi & Truong, 2013; Goss & Lepetit, 2015; Giovagnetti & Ruban, 2018). Some species of Viridiplantae among green algae and non‐vascular plants encode for both proteins in their genome (Alboresi *et al*., 2010; Tibiletti *et al*., 2016). Even if quantitatively the impact of LhcSR is larger in the moss *P. patens* (Alboresi *et al*., 2010), PsbS and LhcSR are both simultaneously active in NPQ activation, with an additive effect, making this species a unique system to compare the molecular mechanisms of the two proteins (Gerotto *et al*., 2012). Moreover, LhcSR and PsbS were both shown to co‐migrate with the *P. patens* PSI–PSII megacomplex when isolated from thylakoid membranes (Furukawa *et al*., 2019).

In this study, we investigated the moss *P. patens zep* KO mutant (Takezawa *et al*., 2015), accumulating Zx constitutively, to assess its role on NPQ activation. The constitutive presence of Zx in the *zep* KO showed a quenched state even in the dark‐adapted state as shown by a remarkable decrease in PSII fluorescence lifetimes *in vivo* and the corresponding reduction in the *F*_v_/*F*_m_ parameter. When light is switched on, the *zep* KO is also faster in triggering an additional quenching.

The generation of *zep psbs* KO and *zep lhcsr* KO mutants allowed us to dissect the individual contributions of PsbS and LhcSR to Zx‐mediated quenching, since the mutation of one protein does not alter the accumulation of the other. PsbS depletion did not alter the dark‐state properties of the *zep* KO, as PSII fluorescence lifetimes and *F*_v_/*F*_m_ values were unchanged between *zep psbs* KO and *zep* KO (Fig. 5). By contrast, LhcSR depletion caused a noticeable increase in the lifetime in the dark compared to *zep* KO, though it remained shorter than in WT. This indicates that LhcSR contributes to the zeaxanthin‐driven constitutive quenching in darkness. Moreover, the *zep psbs lhcsr* KO triple mutant showed no further increase in fluorescence lifetime compared to *zep lhcsr* KO, confirming that this constitutive quenching is entirely dependent on LhcSR, with PsbS playing no significant role in the dark.

Under illumination, NPQ induction was largely unaffected in the *zep* KO in the absence of PsbS alone (see *psbs zep* KO, Fig. 4). However, the lack of LhcSR (*zep lhcsr* KO) abolished the faster NPQ induction seen in *zep* KO, despite high Zx levels, indicating that LhcSR is the primary mediator of this response. Interestingly, the *zep psbs lhcsr* KO mutant exhibited a further reduction in qE after illumination (compared to *zep lhcsr* KO), revealing that PsbS does contribute to NPQ under light, but its activity is less prominent and normally compensated by LhcSR.

Overall, these results elucidated the NPQ mechanism by demonstrating: (1) the presence of two distinct Zx‐dependent quenching components. One that is pH‐independent and in the *zep* KO is activated even in darkness, and a second, light‐dependent component where Zx allosterically enhances LhcSR function; (2) different activation modes of LhcSR and PsbS, with LhcSR contributing to quenching in the dark, while PsbS becomes functionally relevant only under light exposure and amplifies NPQ in the presence of Zx; (3) Zx‐induced quenching does not decrease the PSI lifetime in the dark. It has been reported that PSI might be a site of LhcSR‐dependent NPQ in *C. reinhardtii* (Kosuge *et al*., 2018; Girolomoni *et al*., 2019), but experimental observations showed no differences in PSI excited‐state lifetimes in quenched and unquenched conditions (Tian *et al*., 2017; van Amerongen & Croce, 2025). Our data support the fact that zeaxanthin bound to PSI has no effect on its Chl fluorescence quenching. Finally, PSII lifetime of *zep lhcsr psbs* KO suggested that Zx has residual quenching capacity even in the absence of PsbS and LhcSR.

Concerning the allosteric role of zeaxanthin (Zx) in NPQ regulation, it has been shown that impairment of the xanthophyll cycle can alter LHC conformation (Baroli *et al*., 2003) and the dynamics of protein complexes within thylakoid membranes in both the green alga *C. reinhardtii* (Azadi Chegeni *et al*., 2016; Troiano *et al*., 2021) and vascular plants (Betterle *et al*., 2009; Sacharz *et al*., 2017; Sulli *et al*., 2023). It is worth noting that *zep* KO mutants also lack Nx and Vx (Fig. 1), which may further influence Lhc structural properties, organization, and quenching capacity (Pascal *et al*., 2005; Ruban *et al*., 2007). Indeed, complexes purified from *A. thaliana zep* KO plants retain Zx in the L1 and L2 binding sites of Lhcs (Mozzo *et al*., 2008), whereas in WT plants Zx is mainly bound at the periphery of major and minor antenna proteins (Xu *et al*., 2015). Moreover, different antenna proteins interact with zeaxanthin in distinct ways, reflecting their specific pigment‐binding properties and structural environments. Zx binding to Lhcs such as Lhcb4 and Lhcb5 enhances quenching efficiency in *A. thaliana* (de Bianchi *et al*., 2011; Miloslavina *et al*., 2011), and mutants lacking Lhcb6 show impaired Zx‐dependent NPQ, suggesting that Lhcb6 provides a structural context for Zx‐mediated energy dissipation (Betterle *et al*., 2009). Lhcb6 and other minor antenna proteins are present in *P. patens*, where they may play an important role in Zx‐dependent NPQ.

Since Zx binds to LhcSR *in vivo* (Pinnola *et al*., 2013), we hypothesize that in the *zep KO* background, Zx is incorporated in the LhcSR pigment‐protein complex that regulates the balance between excitation‐energy transfer and excitation‐energy quenching. These findings support *in vivo* a mechanistic model previously proposed based on *in vitro* studies of isolated LhcSR1 complexes (Kondo *et al*., 2017), in which LhcSR can adopt two independently regulated dissipative states, one triggered by lumen acidification and the other by Zx binding. Overall, our results suggest that LhcSR‐dependent NPQ, once fully activated by Zx accumulation, leads to prolonged energy dissipation that persists as long as Zx is present, even in darkness. By contrast, PsbS‐dependent NPQ is strictly light‐dependent, as its activation requires the presence of both Zx and a proton gradient. In this case, Zx does not act directly on PsbS but enhances NPQ by binding to LHC proteins. Considering that Zx epoxidation kinetics generally occur within tens of minutes, LhcSR‐mediated quenching may be advantageous under fluctuating light conditions. This strategy allows for a primed state after a first stress exposure, enabling a faster protective response to subsequent light fluctuations. However, this strategy has also a cost; prolonged NPQ in low light or dark conditions reduces photosynthetic efficiency and may lead to energy loss. Indeed, studies have shown that faster NPQ relaxation combined with faster NPQ induction can positively impact biomass productivity under fluctuating light conditions (Kromdijk *et al*., 2016; De Souza *et al*., 2022).

Thus, while LhcSR is less regulated and may lead to unnecessary energy dissipation under non‐stressful conditions, PsbS introduces a more precise and energy‐efficient mechanism, activating NPQ only when triggered by lumen acidification during light exposure. This shift toward tighter regulation likely drove the evolutionary replacement of LhcSR by PsbS in land plants, enabling NPQ to be more precisely tuned to environmental cues. This hypothesis is supported by our previous observations in *P. patens*, where, during acclimation to fluctuating or high light conditions, PsbS levels increased significantly more than LhcSR (Gerotto *et al*., 2011; Beraldo *et al*., 2025). The evolutionary distribution of LhcSR and PsbS likely reflects not differences in NPQ capacity *per se*, but rather differences in regulatory mechanisms allowing organisms to optimize the balance between photoprotection and photosynthetic efficiency in their specific environments.

## Competing interests

None declared.

## Author contributions

Claudia Beraldo, Cleo Bagchus, DV, AB and CG contributed to the investigation of the study; Claudia Beraldo and AA contributed to the writing – original draft of the study; Claudia Beraldo, TM and AA contributed to the writing – review & editing of the study; EW, HA and AA contributed to the supervision of the study; TM and AA contributed to the conceptualization of the study.

## Disclaimer

The New Phytologist Foundation remains neutral with regard to jurisdictional claims in maps and in any institutional affiliations.

## Supporting information


**Fig. S1** NPQ of WT and *zep* KO mutant corrected for dark‐adapted quenching.
**Fig. S2** *zep psbs* KO mutant isolation.
**Fig. S3** *zep lhcsr1* KO mutant isolation.
**Fig. S4** Multiple *zep lhcsr1 lhcsr2* KO mutant isolation.
**Fig. S5** Multiple *zep psbs lhcsr* KO mutant isolation.
**Fig. S6** Analysis of multiple *zep psbs* KO and *zep lhcsr* KO mutants.
**Fig. S7** Decay‐associated spectra of multiple *zep psbs* KO and *zep lhcsr* KO mutants.
**Table S1** sg‐RNA guides employed to knockout *LhcSR2* gene.
**Table S2** List of primers used in this study.
**Table S3** *F*_v_/*F*_m_ of multiple *psbs* and *lhcsr* and *zep* KO mutants.
**Table S4** Detailed statistical analysis of Fig. 4(e).
Please note: Wiley is not responsible for the content or functionality of any Supporting Information supplied by the authors. Any queries (other than missing material) should be directed to the *New Phytologist* Central Office.

## Data Availability

The data that support the findings of this study are available in the Supporting Information of this article are available in Figs S1, S6, S7; Table S1–S3. The accession numbers associated with the work are the following Pp3c12_10330 (ZEP), Pp3c20_23430 (PSBS), Pp3c9_3440 (LHCSR1), and Pp3c15_11070 (LHCSR2). They were all retrieved from the Phytozome database.
