# Supplementary material for: Functional divergence of LhcSR and PsbS in zeaxanthin‐mediated non‐photochemical quenching
Source: New Phytol. 2026 Apr 11;250(5):3042–52. doi: 10.1111/nph.71176 (PMC13150299; doi:10.1111/nph.71176)
Supplement: Supplementary file 1 — Fig. S1 NPQ of WT and zep KO mutant corrected for dark‐adapted quenching. Fig. S2 zep psbs KO mutant isolation. Fig. S3 zep lhcsr1 KO mutant isolation. Fig. S4 Multiple zep lhcsr1 lhcsr2 KO mutant isolation. Fig. S5 Multiple zep psbs lhcsr KO mutant isolation. Fig. S6 Analysis of multiple zep psbs KO and zep lhcsr KO mutants. Fig. S7 Decay‐associated spectra of multiple zep psbs KO and zep lhcsr KO mutants. Table S1 sg‐RNA guides employed to knockout LhcSR2 gene. Table S2 List of primers used in this study. Table S3 F v/F m of multiple psbs and lhcsr and zep KO mutants. Table S4 Detailed statistical analysis of Fig. 4(e). Please note: Wiley is not responsible for the content or functionality of any Supporting Information supplied by the authors. Any queries (other than missing material) should be directed to the New Phytologist Central Office. [file NPH-250-3042-s001.pdf]

***New Phytologist* Supporting Information**

Article title: Functional Divergence of LhcSR and PsbS in Zeaxanthin-Mediated Non-Photochemical Quenching

Authors: Claudia Beraldo[1], Cleo Bagchus[2], Dana Verhoeven[2], Alessandra Bellan[1], Caterina Gerotto[1,3], Emilie Wientjes[2], Herbert van Amerongen[2], Tomas Morosinotto[1], Alessandro Alboresi[1,4]

Article acceptance date: 15 February 2026

The following Supporting Information is available for this article:

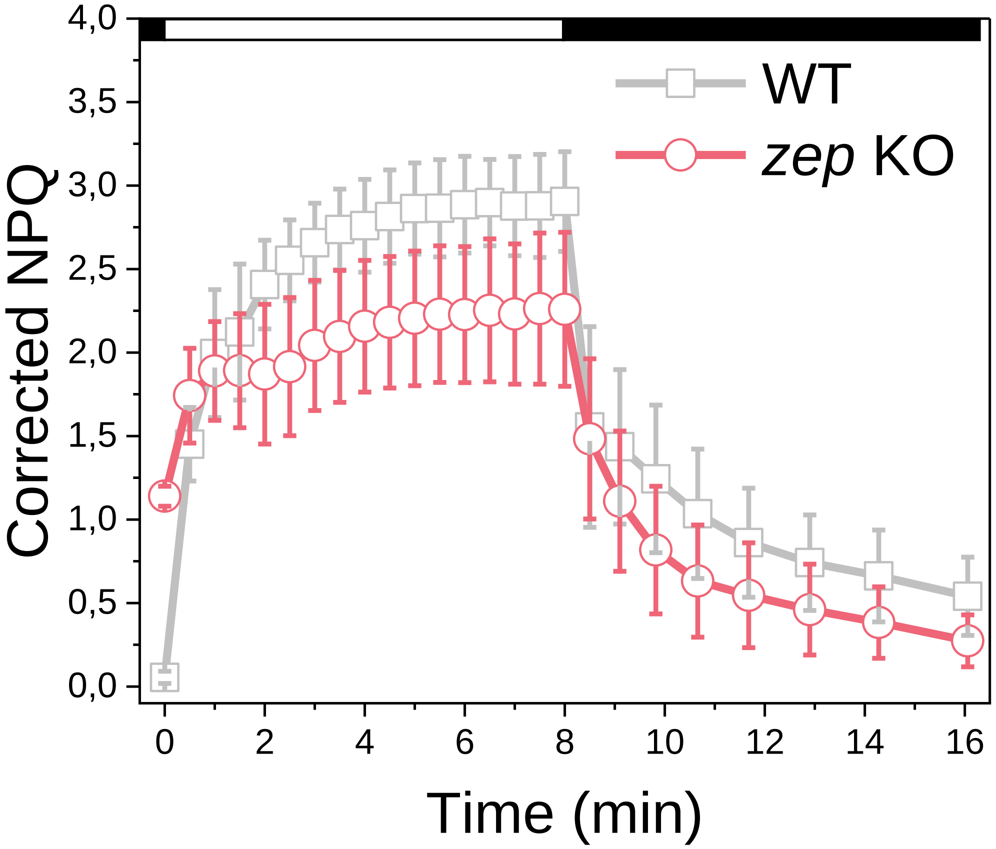

Fig. S1 NPQ of WT and *zep* KO mutant corrected for dark-adapted quenching. NPQ kinetics of WT (grey squares) and *zep* KO (red circles) plants. Exposure to actinic irradiance is indicated at the top of each panel. Actinic light intensity was 850 µmol of photons m^{-2} s^{-1}. The first NPQ data point was obtained for a saturating flash delivered 0.1 seconds after actinic light onset. Data represent mean ± SD from more than four independent biological replicates (n>4).

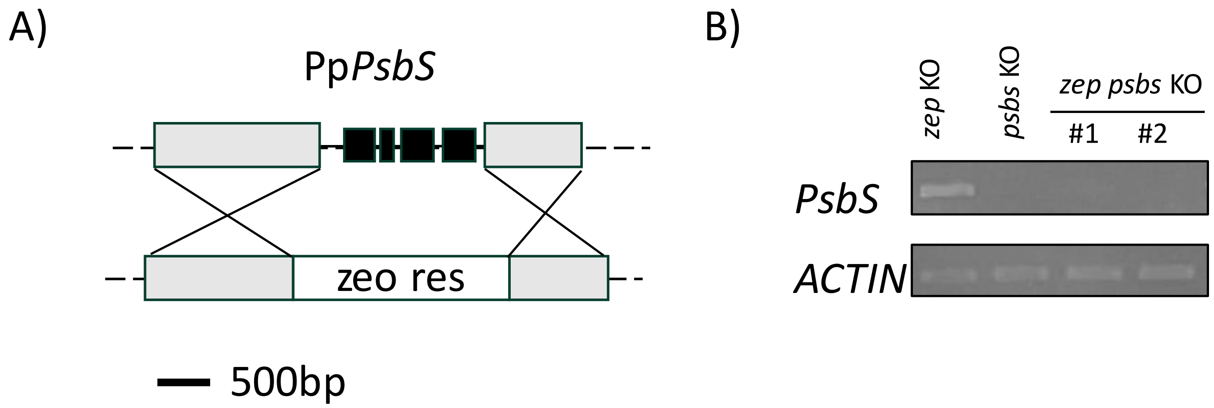

Figure S2. *zep psbs* KO mutant isolation. A) Scheme of construct used for *psbs* KO generation. Simplified representation of genomic region of PsbS. Exons are shown in black, while gray boxes indicate the genomic regions exploited for homologous recombination. Below is shown the constructs for homologous recombination. Zeocine cassette is in between regions homologous the genome. B) RT PCR analysis for the detection of *PsbS* and *ACTIN* transcripts in *zep KO* and two independent putative *zep psbs* KO mosses (clone #1, clone #2).

Click here to enter text.

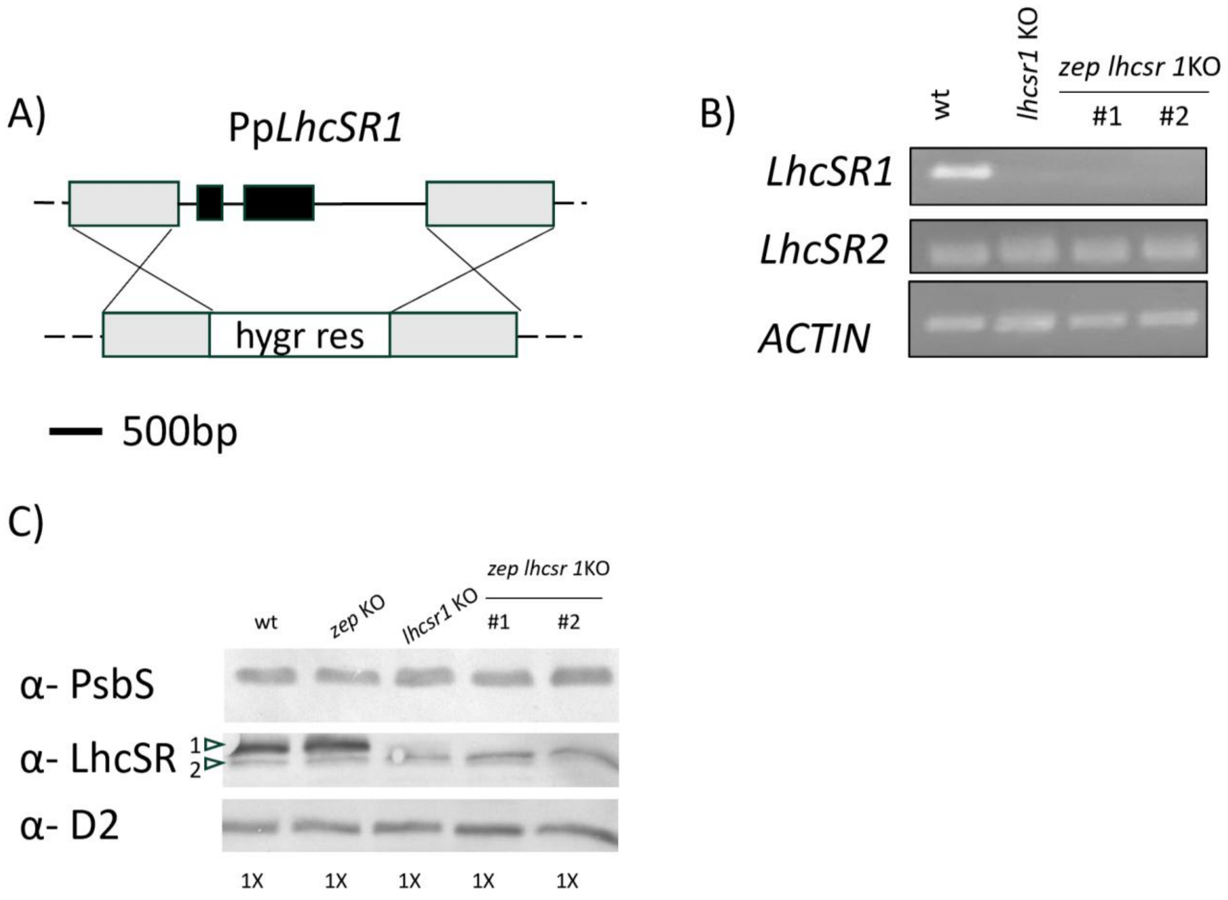

Figure S3. *zep lhcsr1* KO mutant isolation. A) Scheme of construct used for *lhcsr1* KO generation. Simplified representation of genomic region of LhcSR. Exons are shown in black, while grey boxes indicate the genomic regions exploited for homologous recombination. Below is shown the constructs for homologous recombination. Hygromycin cassette is located between regions homologous the genome. B) RT PCR analysis for the detection of *LhcSR1* and *LhcSR2* and *ACTIN* transcripts in *zep KO* and two independent putative *zep lhcsr1* KO mosses (clone #1, clone #2). C) Immunoblot analysis for the detection of PsbS, LhcSR and D2 proteins in WT, *zep* KO, *lhcsr1* KO and two putative *zep lhcsr1* KO mosses (clone #1, clone #2). 1X is equivalent to 0.5µg of chlorophyll Immunoblot of WT, *zep* KO, *lhcsr1* KO, *zep lhcsr*1 KO (clone #1, clone #2).

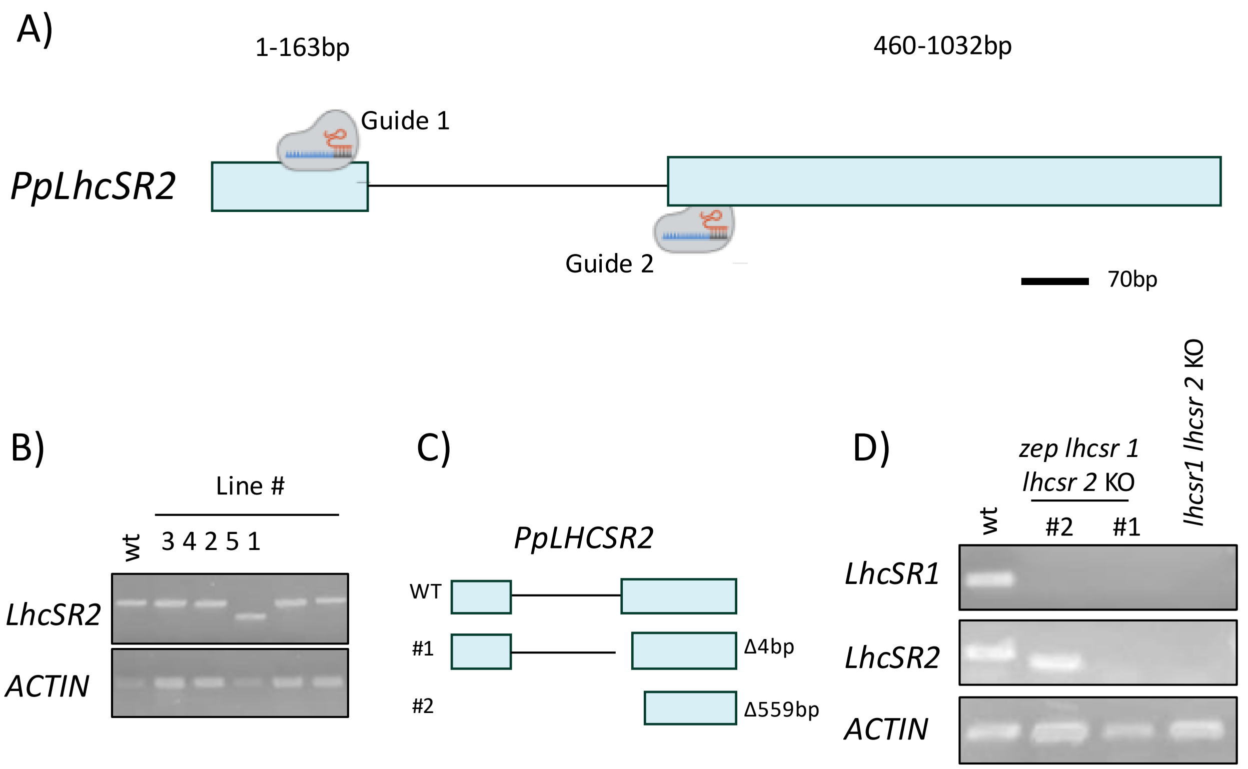

Figure S4. Multiple *zep lhcsr1 lhcsr2* KO mutant isolation. A) Methods. Blue boxes indicate exon 1 and exon 2 of *LhcSR2* gene. Black line intron. sg-RNA guide 1 and sg-RNA guide 2 were designed to target respectively exon 1 and exon 2. B) Example of PCR analysis on genomic DNA for the detection of *LhcSR2* and *ACTIN* genes in WT and independent putative *zep lhcsr1 lhcsr2* KO mosses indicated with different numbers. C) Sequencing results of selected lines. Clone#1 showed 4bp deletions in the second exon. Clone#2 showed deletions of exon 1, intron and part of exon 2. D) RT-PCR for the detection of *LhcSR1*, *LhcSR2* and *ACTIN* transcripts in WT, *lhcsr1/2* KO and selected *zep lhcsr1/2* KO lines.

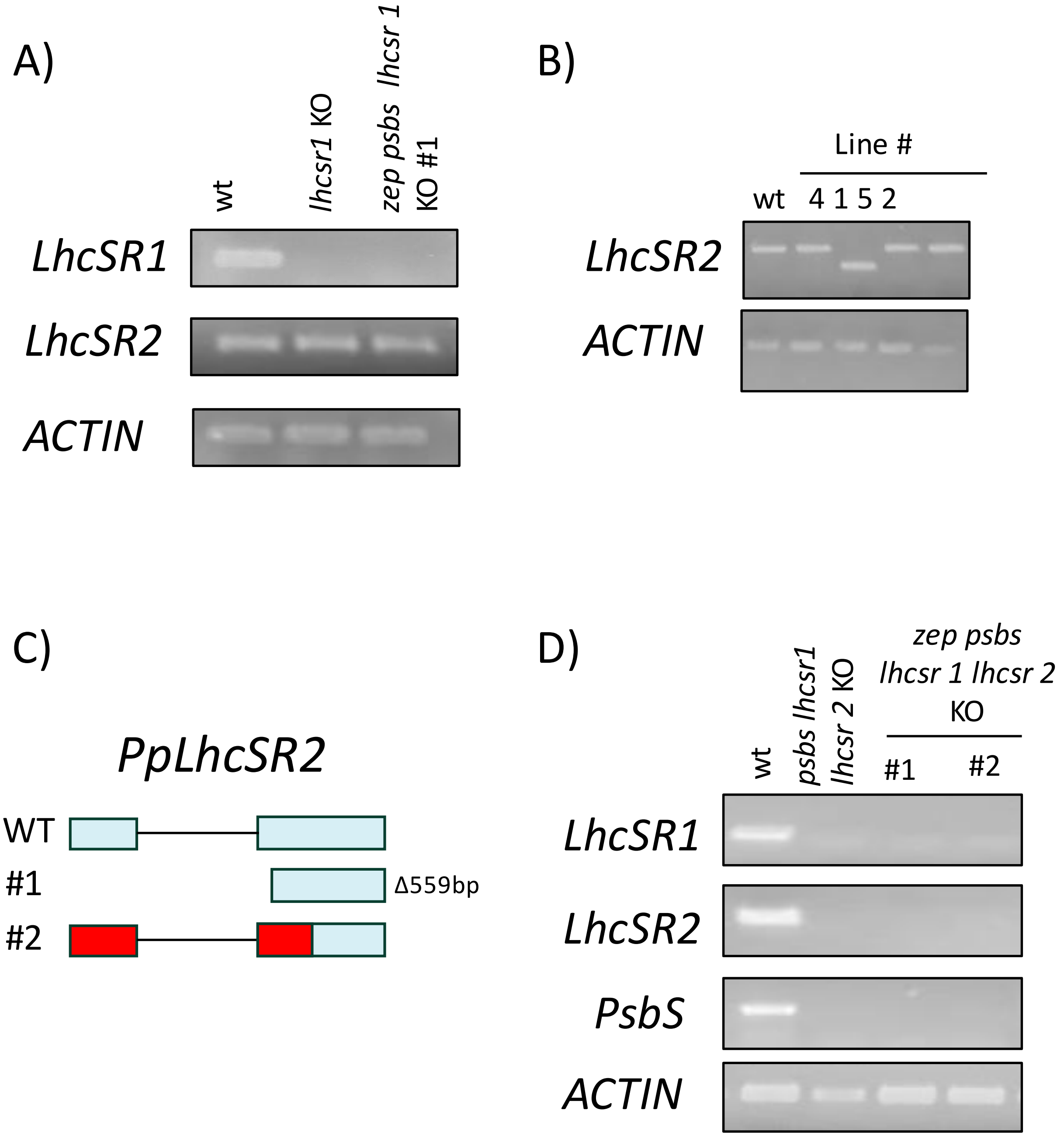

Figure S5. Multiple *zep psbs lhcsr* KO mutant isolation. A) RT-PCR for the detection of *PsbS, LhcSR1*, *LhcSR2* and *ACTIN* transcripts in WT and *zep psbs lhcsr1* KO line. B) Example of PCR analysis on genomic DNA for the detection of *LhcSR2* and *ACTIN* genes in WT and independent putative *zep psbs lhcsr1/2* KO mosses indicated with different numbers. B) Sequencing results of selected lines. Clone#1 showed 559bp deletions in the second exon. Clone#2 showed mismatches at level of exon 1, intron and part of exon 2 (red boxes). C) RT-PCR for the detection of *PsbS, LhcSR1*, *LhcSR2* and *ACTIN* transcripts in WT and selected *zep lhcsr1/2* KO lines.

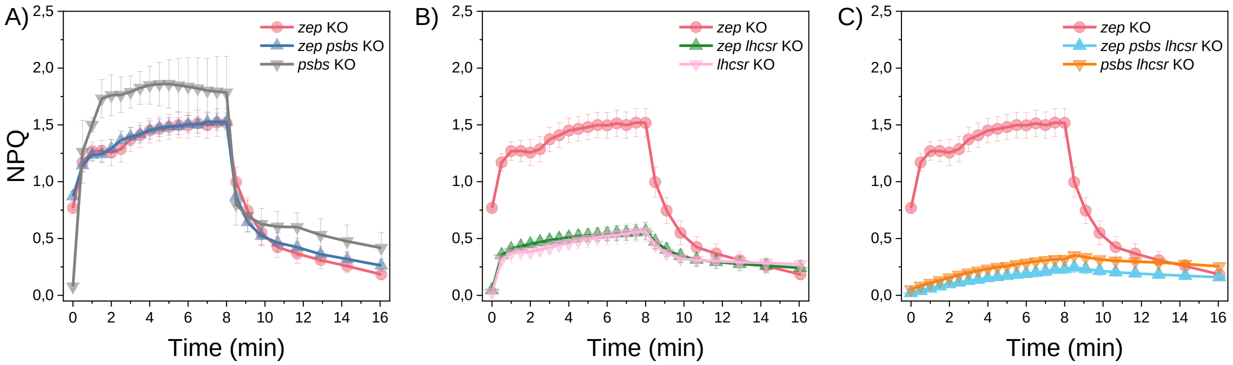

Figure S6. Analysis of multiple *zep psbs* KO and *zep lhcsr* KO mutants. A-C) NPQ kinetics of *zep* KO (red) *zep psbs* KO (blue), *psbs* KO (grey), *zep lhcsr* KO (green), *lhcsr* KO (pink), *zep psbs lhcsr* KO (light blue), *psbs lhcsr* KO (orange). 10 days old protonema was treated with 850 µmol of photons m^{-2} s^{-1}. Data represent mean ± SE (*psbs* KO, *lhcsr* KO, *psbs* KO n=3, *zep*, *zep psbs*, *zep lhcsr*, *zep psbs lhcsr* n=5).

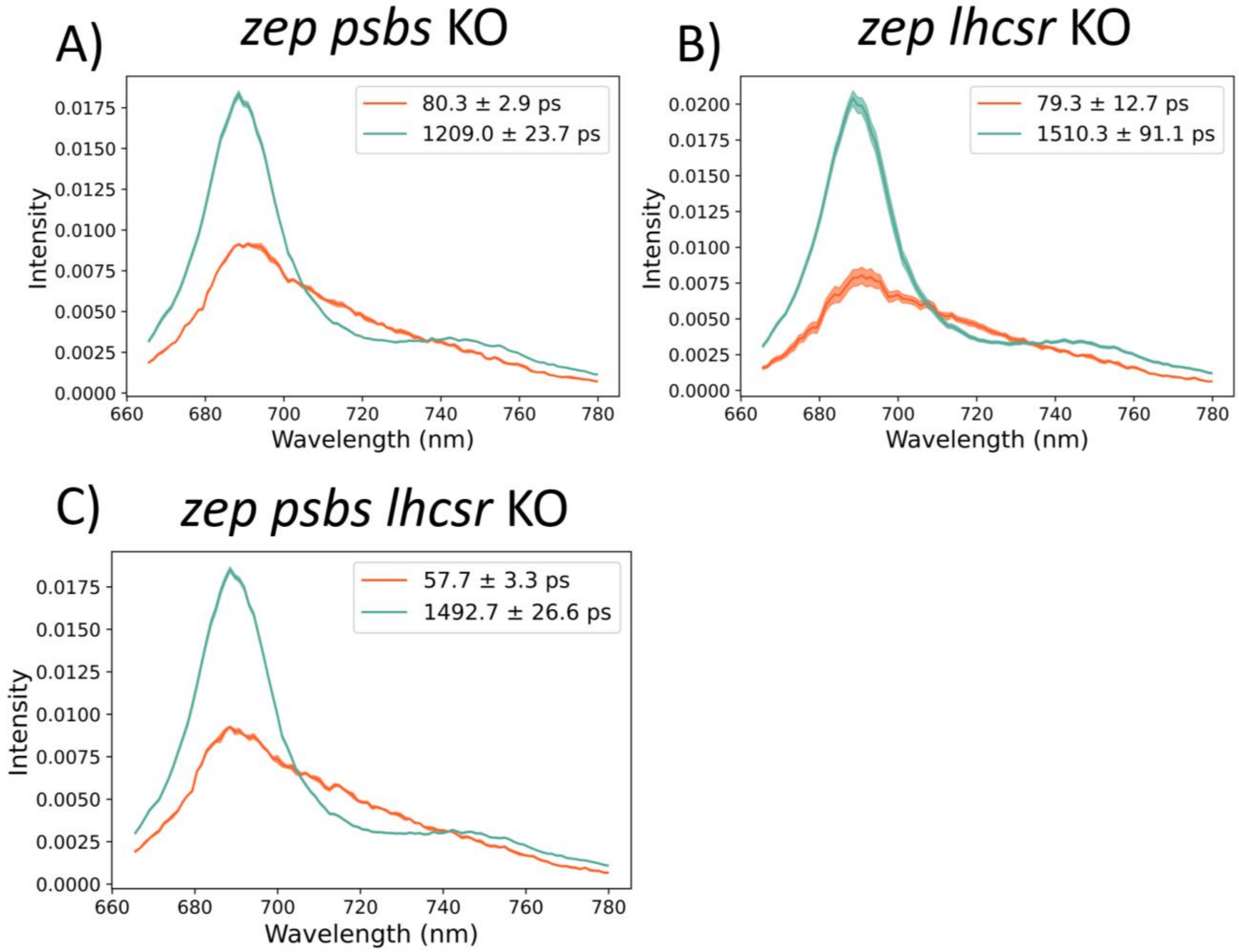

Figure S7. Decay-associated spectra of multiple *zep psbs* KO and *zep lhcsr* KO mutants. A-C) *zep psbs* KO, *zep lhcsr* KO, *zep psbs lhcsr* KO. The spectra are measured in F_m conditions (closed reaction centers with DCMU). The green spectrum is attributed to PSII and the orange spectrum is attributed to PSI. Shading indicates the standard deviation. The fluorescence lifetimes ± SD are indicated in the insets (n=3).

Target	sg-RNA guide
*lhcsr*2-exon 1	CAGCTGCACCTCACGCAACG
*lhcsr2-e*xon 2	TCGAACGGAGCAGTCACTCC

Table S1. sg-RNA guides employed to knock out *LhcSR2* gene.

Primer	Use	Sequence
PsbS FOR	RT-PCR	CTTTGGGTGACCGTGGAA
PsbS REV	RT-PCR	ACACGGGGCCCTTTTCAT
LhcSR1 FOR	RT-PCR	CCCCAGCAGCCTACCAGGTG
LhcSR1 REV	RT-PCR	AGGCAGCACGAGCCAACAAA
LhcSR2 FOR	RT-PCR	TTGGATCTTAGAAGTGCAAGAAA
LhcSR2 REV	RT-PCR	TTGCAGCTGCACCTCACGCA
ACTIN FOR	RT-PCR/KO screening	GCGAAGAGCGAGTATGACGAG
ACTIN REV	RT-PCR/KO screening	AGCCACGAATCTAACTTGTGATG
LhcSR2 FOR	KO screening	TCCCACTGTGTTGTCCTCTC
LhcSR2 REV	KO screening	AGCTTCGGTAAACTCAGCCT

Table S2. List of primers used in this study.

Genotype	F_v/ F_m	Tukey HSD Label
WT	0,77±0,02	a
psbs KO	0,77±0,03	a
lhcsr KO	0,77±0,02	a
psbs lhcsr KO	0,78±0,02	a
zep KO	0,69±0,03	b
zep psbs KO	0,69±0,02	b
zep lhcsr KO	0,73±0,02	ab
zep lhcsr psbs KO	0,74±0,02	ab

Table S3. F_v/F_m of multiple *psbs* and *lhcsr* and *zep* KO mutants. Values labelled with different letters are significantly different (One-Way ANOVA followed by TukeyHSD post-hoc test, $p < 0.01$, $n>3$).

One Way Anova-0,1s	N Analysis	N Missing	Mean	Standard Deviation	SE of Mean	Tukey-Post Hoc Label
WT	5	0	0,0546	0,03662	0,01638	b
zep KO	7	0	0,76557	0,04002	0,01513	a
zep psbs KO	8	0	0,87188	0,12275	0,0434	a
zep lhcsr KO	5	0	0,046	0,01651	0,00738	b
zep psbs lhcsr KO	4	0	0,021	0,01349	0,00675	b
**Overall ANOVA**	**DF**	**Sum of Squares**	**Mean Square**	**F Value**	**Prob>F**	
Model	4	44,543	111,358	21,889,931	<0.0001	
Error	24	0,12209	0,00509			
Total	28	457,639				
**Fit statistics**	**R-Square**	**Coeff Var**	**Root MSE**	**Data Mean**		
	0,97332	0,16008	0,07132	0,44555		

One Way Anova-8min	N Analysis	N Missing	Mean	Standard Deviation	SE of Mean	Tukey-Post Hoc Label
WT	5	0	29,044	0,29882	0,13364	a
zep KO	6	1	1,622	0,15303	0,06247	b
zep psbs KO	8	0	143,725	0,30714	0,10859	b
zep lhcsr KO	5	0	0,5648	0,16949	0,0758	c
zep psbs lhcsr KO	4	0	0,2305	0,08118	0,04059	c
**Overall ANOVA**	**DF**	**Sum of Squares**	**Mean Square**	**F Value**	**Prob>F**	
Model	4	2,057,845	514,461	9,322,309	<0.0001	
Error	23	126,928	0,05519			
Total	27	2,184,772				
**Fit statistics**	**R-Square**	**Coeff Var**	**Root MSE**	**Data Mean**		
	0,9419	0,16653	0,23492	141,064		

Table S4. Detailed statistical analysis of Figure 4E.
